# Prevalence and risk factors associated with HIV/hepatitis B and HIV/hepatitis C co-infections among people who inject drugs in Mozambique

**DOI:** 10.1186/s12889-020-09012-w

**Published:** 2020-06-03

**Authors:** Cynthia Semá Baltazar, Makini Boothe, Timothy Kellogg, Paulino Ricardo, Isabel Sathane, Erika Fazito, Henry F. Raymond, Marleen Temmerman, Stanley Luchters

**Affiliations:** 1 Instituto Nacional de Saúde (INS), Maputo, Mozambique; 2 Department of Public Health and Primary Care, Faculty of Medicine and Health Sciences, Ghent University, Ghent, Belgium; 3 University of California, San Francisco, USA; 4 National Program to Control STIs and HIV/AIDS, Ministry of Health, Maputo, Mozambique; 5 International Center Aids Program (ICAP), Maputo, Mozambique; 6 School of Public Health, Rutgers University, Piscataway, NJ USA; 7 Department of OBGYN, Aga Khan University, Nairobi, Kenya; 8 Department of Population Health, Aga Khan University, Nairobi, Kenya; 9 Department of Epidemiology and Preventive Medicine, Monash University, Melbourne, Australia; 10 Burnet Institute, Melbourne, Australia

**Keywords:** People who inject drugs, HIV, Hepatitis B, Hepatitis C, Co-infections, Respondent driven sampling, Mozambique

## Abstract

**Background:**

There is scare information about HIV co-infections with hepatitis B virus (HBV) and/or hepatitis C virus (HCV) among People Who Inject Drugs (PWID) in Mozambique. This information is critical to ensure the treatment necessary to decrease the progression of liver disease and the transmission of both HIV and hepatitis. We assess the prevalence of HIV, HBV and HCV co-infections as well as associated risk factors among PWID.

**Methods:**

The first Bio-Behavioral Surveillance Survey was conducted in 2013–2014 among persons who self-reported to have ever injected drugs. Using respondent-driven sampling, PWID aged 18 years and older were recruited in two cross-sectional samples in Maputo and Nampula/Nacala, two large urban centers of Mozambique. Rapid screening of HIV, HBV (HBsAg) and HCV was performed on site. Data from participants in both cities were pooled to conduct RDS-weighted bivariate analyses with HIV/HBV and HIV/HCV co-infections as separate outcomes. Unweighted bivariate and multivariate logistic regression analyses were conducted to assess correlates of co-infection.

**Results:**

Among 492 eligible PWID, 93.3% were male and median age was 32 years [IQR: 27–36]. HIV, HBV and HCV prevalence were respectively 44.9% (95% CI:37.6–52.3), 32.8% (95% CI:26.3–39.5) and 38.3 (95% CI:30.6–45.9). Co-infections of HIV/HBV, HIV/HCV and HIV/HBV/HCV were identified in 13.1% (95% CI:7.2–18.9), 29.5% (95% CI:22.2–36.8) and 9.2% (95% CI:3.7–14.7) of PWID, respectively. Older age, history of needle/syringe sharing and history of injection with used needle/syringe was associated with HIV/HBV co-infection. Living in Maputo city, have older age, history of needle/syringe sharing and history of injection with used needle/syringe was associated with HIV/HCV co-infection.

**Conclusion:**

There is a high burden of HBV and HCV among HIV-infected PWID in Mozambique. Our results highlight the need for targeted harm reduction interventions that include needle exchange programs and integrated services for the diagnosis and treatment of HIV, HBV and HCV to address these epidemics among PWID. Efforts should be made to strengthen ART coverage in the population as an important treatment strategy for both viruses.

## Background

People who Inject Drugs (PWID) are among the most vulnerable to HIV infection, with an estimated prevalence of 17.8% globally and 8.3% in the Sub-Saharan African region [1]. PWID are considered a key population for targeted HIV prevention and treatment efforts due to their illicit drug use and associated social stigma, which often makes it difficult to obtain information about this population and increases their vulnerability to HIV transmission [2, 3].

HIV ranks among one of the highest HIV prevalence rates among adults aged 15–49 years old age (13.2%), largely due to heterosexual transmission [4]. Although the 3rd National HIV Strategic Plan recognized PWID as a key population in 2010, there has been a lack of information about the population size, HIV prevalence and their utilization of health services [5]. To address this gap, the first Bio-Behavioral Surveillance (BBS) survey among PWID was conducted in two main urban cities between 2013 and 2014, using respondent-driven sampling (RDS). This study estimated HIV prevalence at 50.1% in Maputo (95% CI: 40.1–59.0) and 19.9% in Nampula/Nacala (95% CI: 10.9–29.2), where the greater burden of HIV observed in the southern region is consistent with the dynamics of the HIV epidemic in the country [6, 7],.

In addition to HIV, viral hepatitis remains an important global health issue, characterized by high prevalence, high burden of morbidity and mortality, and suboptimal diagnosis and management approaches [8–10]. Viral hepatitis are the most common viral infections affecting PWIDs [10, 11]. HIV and viral hepatitis infections share common transmission routes, risk behaviors and their co-infection represents a serious global concern resulting in high morbidity and mortality rates [11, 12]. HIV infection affects the natural course of hepatitis B virus (HBV) and hepatitis C virus (HCV), promoting faster progression to chronicity, liver fibrosis, and malignancy [2, 9–11]. The BBS among PWID in Mozambique estimated HBV prevalence at 32.1 and 36.4%, and HCV prevalence at 44.6 and 7.0%, in Maputo and Nampula/Nacala respectively. Data about HBV and HCV among the general population in Mozambique is limited to a few studies in the southern region of the country [13, 14].

Global health targets to reduce HIV and viral hepatitis infections have been established [15, 16]. Strengthening the prevention and treatment of substance abuse, including narcotic drug abuse, is listed as one of the priorities of the United Nations 2030 Agenda for Sustainable Development Goals (Goal 3.5) [17]. Integrated harm reduction programs, in some countries have been designed to address the assumption that the prevention of HIV in PWID will also prevent hepatitis given their similar modes of transmission and high-risk populations.

PWID have the highest burden of HIV among key population groups in Mozambique and yet there continues to be lack of integrated prevention and treatment services [18, 19]. In a country with one of the highest HIV prevalence rates in the world, understanding the epidemiology of HIV, HBV and HCV among PWID is critical in order to design effective and efficient prevention and treatment programs. Neglecting to implement targeted services has the potential to compromise the success of the overall HIV response in the country.

As such, the objective of this secondary analysis was to evaluate HIV/HBV and HIV/HCV co-infections among PWID in Mozambique and to assess associated risk factors. The findings from this paper emphasize the importance of focusing on the dynamics of this population and could also support the current drafting of the National Harm Reduction Plan. The population has the highest burden of HIV among key population groups and yet there continues to be lack of integrated services for HIV and viral hepatitis. Since Mozambique has one of the highest HIV prevalence rates in the world, neglecting to implement targeted services has the potential to compromise the success of HIV prevention and control programs in the country. The findings from this paper emphasize the importance of focusing on the dynamics of this population and could also support the current drafting of the National Harm Reduction Plan.

## Methods

### Study design, target population, recruitment, and sampling

We conducted a cross-sectional survey using RDS sampling methodology in the two urban cities of Maputo and Nampula/Nacala, located in the South and North regions of Mozambique, respectively. This survey methodology has been widely used among hard-to-reach populations to estimate HIV prevalence and associated risk factors, and also to evaluate access and use of health services [6, 7, 20–25]. Recruitment occurred between September 2013 and March 2014. The recruitment procedures have been published previously [6, 7].

Participants were be eligible for inclusion in the study, if they were at least 18 years of age; lived, worked or socialized in the survey area within the last 12 months; injected drugs without a medical prescription within the last 12 months (in December 2013, eligibility requirements were expanded to anyone who had ever injected drugs at any point in their lives to overcome recruitment challenges). They also needed to be in possession of a valid recruitment coupon and have and mental ability to provide written informed consent. Participants received a prevention and hygiene kit, 50 MZN (~$2.50) in cash to compensate for transportation costs, and 50 MZN worth of mobile phone credit for every successfully referred peer who participated in the survey.

A behavioral questionnaire was administered, and blood sample collected for on-site rapid tests for HIV, HBV and HCV. The HIV rapid testing procedures adhered to the national algorithm using Determine™ HIV-1/2 (Alere, USA), followed by a confirmatory test using Uni-Gold HIV™ (Trinity Biotech, Ireland). The results of the rapid HIV tests, together with self-reported prior HIV-positive test results, were used to estimate HIV prevalence. On-site rapid HBV testing was done using Alere Determine® HbsAg (Abbott Laboratories, UK), to detect surface antigen, and HCV testing used SD Bioline HCV (Standard Diagnostics, Korea). Participants with reactive HbsAg results or HCV antibodies were considered as having an active HBV and HCV infection, respectively; none of the tests were able to determine if infection was acute or chronic. Participants with positive results for any of the laboratory tests were referred to a nearby health facility, identified during the formative assessment, where trained teams were ready to link them to treatment services [6, 7].

*Dried Blood Spot (DBS)* were also collected and analyzed at the central laboratory of the National Institute of Health. Central-level HBV tests were performed at the National Institute of Health Laboratory using ELISA Murex® HBsAg Version 3 (Murex Biotech Limited, UK) and were used to estimate HBV prevalence in the survey; centralized HCV testing was not performed because a serologic anti-HCV assay using DBS had not been validated by the National Reference Laboratory at the time of the survey [6, 7].

Separate informed consent was required for behavioral questionnaire and each separate laboratory test; 47 participants did not consent to rapid HIV testing, two participants did not consent for HBV, and 46 participants did not consent to HCV testing.

### Behavioral indicators

Demographic information and self-reported sexual and injection risk behaviors were recorded through face-to-face interviews using a structured questionnaire delivered by trained interviewers. The network size was assessed by asking: “Approximately how many people who inject drugs do you think live in and around the city of <Maputo or Nampula>?”

### Statistical analysis

For the purpose of this analysis, data on survey participants from both cities were pooled given the low sample size. RDS-adjusted pooled descriptive statistics were used to describe participants demographic characteristics, drug use behaviors, HIV/HBV, HIV/HCV, HIV/HBV/HCV co-infections, as well as singular infections. Site level analysis of these variables were adjusted for the sampling method where the adjustment takes into consideration the probability of each participants inclusion in the study based on their self-reported network size. Pooled estimates were weighted by the size of the PWID population in each study site, based on four approaches to produce estimates of the PWID population size in each city. The median of the four estimates in each site resulted in a PWID population size of 1684 in Maputo and 520 in Nampula. These RDS-adjusted pooled prevalence estimates and 95% confidence intervals (CI) were obtained using the *aggregate estimate* feature within the RDS Analyst suite of tools [26].

Unadjusted pooled bivariate and multivariable logistic regression analyses were conducted to identify correlates for the two outcomes of interest: HIV/HBV and HIV/HCV. Correlates included in the final model were selected based on literature review and the results of the bivariate association (*p* <  0.05) with the outcomes of interest. This analysis was conducted using R Statistical Software v.3.1.1 (r Development Core Team, Vienna, Austria).

### Ethical considerations

This is a secondary data analysis from a survey protocol that was approved by the Mozambican National Bioethics Committee for Health (CNBS) (46/CNBS/13) and the Institutional Review Board of the University of California at San Francisco (13–10,699); the US Centers for Disease Control and Prevention (CDC) determined the protocol to be research where CDC was not engaged. Participants provided informed consent for study participation and no personal identifying information was collected.

## Results

### Population characteristics

A total of 353 PWID were enrolled in the survey in Maputo and 139 in Nampula/Nacala. When pooling results, the majority of participants were male (93.3, 95%CI: 90.3–96.3), and the median age was 32 years. Half (49.9, 95%CI: 42.8–55.9) had completed secondary or higher education. The majority of participants were Christian (54.8, 95%CI: 48.3–61.3) and were single or never married (60.9, 95%CI: 55.6–66.3). Table 1 presents the RDS-Adjusted characteristics of the survey participants.

**Table 1:** People who Inject Drugs population description in two urban settings in Mozambique, 2013–2014 (*N* = 492)

Characteristics	PWID Study Population		
	***n***	% unweighted	RDS-weighted % (95%CI)
**City**			
Maputo	353	71.7	**–**
Nampula/Nacala	139	28.3	**–**
**Crude median age (years) [IQR]**^**a**^	32 [27–36]		
**Age group (years)**			
18–24	92	18.7	18.2 (1.3–23.3)
≥ 25	400	81.3	81.8 (76.7–86.8)
**Gender**			
Male	467	94.9	93.3 (90.3–96.3)
Female	25	5.1	6.7 (3.6–9.7)
**Marital status**			
Single/never married	289	58.7	60.9 (55.6–66.3)
Cohabitating/married	103	20.9	20.5 (13.9–26.9)
Widowed/divorced/separated	100	20.3	18.6 (13.1–24.1)
**Education**			
No formal education/primary	210	42.8	50.1 (44.0–57.2)
Secondary/higher	281	57.2	49.9 (42.8–55.9)
**Religion**			
Christian (catholic, evangelical, protestant)	247	50.2	54.8 (48.3–61.3)
Muslim	143	29.1	25.5 (19.5–31.5)
Other/None	102	20.7	19.7 (14.4–24.9)
**Age of first drug use (years)**			
< 18	56	11.7	7.9 (0.1–14.9)
18–24	235	49.1	50.9 (45.8–55.9)
≥25	188	39.2	41.2 (34.5–47.8)
**No of sexual partners, last 12 months, males only (*N*** **= 456)**^**b**^			
0	94	20.6	19.9 (15.5–24.3)
1	95	20.8	24.2 (19.0–29.4)
≥2	267	58.6	55.9 (49.6–62.1)
**Frequency of drug injection in the last 12 months**			
Did not use	25	5.1	7.9 (4.5–11.8)
Few times per year or month	192	39.0	40.0 (34.0–46.1)
Daily	275	55.9	51.9 (45.4–58.4)
**Had access to new syringes in the past 12 months (*N*** **= 491)**	425	86.6	87.3 (82.6–92.1)
**Ever shared needles or syringes**	258	52.4	48.8 (42.1–55.6)
**Shared syringes in the last month**	146	29.7	27.2 (20.5–33.8)
**Ever injected with a previously used needle/syringe (*N*** **= 491)**	391	79.6	74.8 (68.6–81.1)
**Ever shared any other injection equipment**^**c**^	158	32.1	33.6 (26.9–40.4)
**Had unprotected sexual intercourse at last sex (N = 491)**			
Yes	214	43.6	44.3 (37.6–51.0)
No, used a condom	173	35.2	36.1 (30.2–42.0)
Not sexually active	104	21.2	19.4 (13.1–25.8)
**Received condoms in the past 12 months (*N*** **= 489)**	188	38.4	34.6 (28.6–40.6)
**Ever incarcerated**	328	66.7	64.3 (58.2–70.6)

^a^ IQR Interquartile range

^b^ 11 male subjects did not report number of sexual partners

^c^ Other includes spoon, cotton or cleaning agent

About half the participants (50.9, 95% CI: 45.8–55.9) reported initiation of injection drug use when they were between the ages of 18–24 years and reported daily drug injection during the past year (51.9, 95%CI: 45.4–58.4). Although 87.3% (95%CI: 82.6–92.1) of participants stated that they had access to brand new syringes, sharing was a common practice and 48.8% (95%CI: 42.1–55.6) of participants reported having shared a needle or syringe at least once in their lifetime, while a quarter (27.2, 95%CI: 20.5–33.8) reported sharing in the month prior to the survey. Nearly two-thirds of participants (64.3, 95%CI: 58.2–70.6) reported that they have ever been incarcerated (Table 1).

### Prevalence of HIV, HBV, HCV and co-infections

As presented in Table 2, HIV prevalence among participants was 44.9% (95% CI: 37.6–52.3). The prevalence of HBsAg among PWID was 32.8% (95%CI: 26.3–39.5) and 38.3% for hepatitis C virus antibody (95%CI: 30.6–45.9). Co-infection of HIV/HBV was found in 13.1% (95%CI: 7.2–18.9), and HIV/HCV co-infection was found in 29.5% (95%CI: 22.2–36.8) of PWID. Triple infection of HIV/HBV/HCV was identified in 9.2% (95%CI: 3.7–14.7) of PWID.

**Table 2:** Prevalence of HIV, hepatitis and co-infections in PWID in two urban settings in Mozambique, 2013–2014

Characteristics	PWID Study Population		
	***n***	% unweighted	RDS-weighted % (95%CI)
**HIV infected** (*N* = 445^a^)	204	45.8	44.9 (37.6–52.3)
**HBV infected** (*N* = 490^a^)	164	33.5	32.8 (26.3–39.5)
**HCV infected** (*N* = 446^a^)	163	36.5	38.3 (30.6–45.9)
**HIV/HBV co-infection** (*N* = 444^a^)	59	13.3	13.1 (7.2–18.9)
**HIV/HCV co-infection** (*N* = 424^a^)	124	29.2	29.5 (22.2–36.8)
**HIV/HBV/HCV co-infection** (*N* = 423^a^)	40	9.5	9.2 (3.7–14.7)

^a^ Denominator reflects the number of participants who consented to testing for each biomarker

### Associated risk factors for HIV/HBV and HIV/HCV co-infection

In multivariate analysis, older age (aOR 12.9, 95%CI: 3.6–233.5), ever shared needles/syringes (aOR 1.8, 95%CI: 1.2–3.4) and ever injected with a previously used needle/syringe (aOR 5.3, 95%CI: 1.9–22.4) was associated with HIV/HBV co-infection (Table 3). Living in Maputo (aOR 4.4, 95%CI: 2.0–11.1), older age (aOR 22.8, 95% CI: 4.6–414.6), ever sharing needles or syringes (aOR 4.0, 95%CI 2.1–7.7), ever injecting with a previously used needle/syringe (aOR 12.1, 95%CI: 4.1–46.3) were associated with HIV/HCV co-infection (Table 4).

**Table 3:** Predictors of HIV/HBV co-infection among PWID study participants, 2013–2014 (*N* = 442)

Characteristics	n/N	Prevalence of HBV/HIV	Crude OR	95% CI	***p***-value	aOR	95% CI	p-value
**City**								
Maputo	49/318	15.1	2.1	0.9–3.9	0.01	1.5	0.7–3.4	0.27
Nampula/Nacala	10/126	7.9	1					
**Age group (years)**								
18–24	1/83	1.2	1					
≥25	58/361	16.1	15.7	3.4–279.5	< 0.01	12.9	2.6–233.5	< 0.01
**Gender**								
Male	56/424	13.2	0.9	0.3–3.8	0.17	–	–	–
Female	3/20	15.0	1					
**Marital status**								
Single/never married	33/260	12.7	0.8	0.4–1.6	0.52	–	–	–
Cohabitating/married	12/93	12.9	0.8	0.3–1.8	0.63	–	–	–
Widowed/divorced/separated	14/91	15.4	1					
**Education level**								
No formal education/Primary school	22/189	11.6	0.7	0.4–1.3	0.37	–	–	–
Secondary/Higher	37/254	14.6	1					
**Age at first drug use (years)**								
< 18	6/50	12.0	1					
18–24	29/213	13.6	1.2	0.4–3.2	0.76	–	–	–
≥25	22/169	13.0	1.1	0.4–3.1	0.85			
**No of sexual partners, last 12 months**								
0	10/89	11.2	1					
1	11/85	12.9	1.7	0.4–2.9	0.73	–	–	–
≥2	35/239	14.6	1.3	0.6–3.0	0.43	–	–	–
**Gave money, good goods or services in exchange for sex in the, past 12 months**^**a**^								
Yes	19/182	10.4	0.6	0.3–1.1	0.10	–	–	–
No	37/231	16.0	1					
**Received money, goods or services in exchange for sex, past 12 months**								
Yes	7/73	9.6	0.6	0.3–1.3	0.27	–	–	–
No	49/340	14.4	1					
**Ever received drugs in exchange for sex, lifetime**								
Yes	10/59	16.9	1.3	0.6–2.8	0.38	–	–	–
No	49/384	12.8	1					
**Had unprotected sexual intercourse at last sex**								
Yes	22/196	11.2	1.0	0.47–2.2	0.18	–	–	–
No, used a condom	26/149	15.0	1.7	0.08–3.7	1.01	–	–	–
Not sexually active	11/98		1					
**Frequency of drug use, last 12 months**								
Did not use	1/22	4.3	1					
Daily	38/249	15.3	3.9	0.7–71.9	0.18	–	–	–
Few times per year/month week	20/172	11.6	2.8	0.6–53.2	0.31	–	–	–
**Ever shared needles/syringes**								
Yes	42/244	17.2	2.2	1.3–4.2	< 0.01	1.8	1.2–3.4	0.05
No	17/200	8.5	1					
**Shared needles/syringes in the last month**								
Yes	22/138	15.9	1.3	0.7–2.4	0.27	–	–	–
No	37/306	12.1	1					
**Ever injected with a previous used needle/syringe**								
Yes	56/358	15.6	5.0	1.8–21.1	< 0.01	5.3	1.9–22.4	0.01
No	3/85	3.5	1					
**Ever shared any other injection equipment**^**b**^								
Yes	18/146	12.3	0.8	0.4–1.5	0.64	–	–	–
No	35/250	14.0	1					
**Had access to new syringes in the past 12 months**								
Yes	51/382	13.4	1					
No	7/61	11.5	0.8	0.3–1.8	0.68	–	–	–
**Received condoms in the past 12 months**								
Yes	27/172	15.7	1					
No	32/269	11.9	0.7	0.4–1.2	0.25	–	–	–
**Ever incarcerated**								
Yes	45/296	15.2	1.7	0.9–3.3	0.09	1.2	0.6–2.4	0.63
No	14/148	9.5	1					

^a^ Excluded female participants

^b^ Other includes spoon, cotton or cleaning agent

**Table 4:** Correlates of HIV/HCV co-infections among PWID study participants, 2013–2014 (*N* = 422)

Characteristics	n/N	Prevalence of HCV/HIV	OR	95%CI	***p***-value	aOR	95% CI	***p***-value
**City**								
Maputo	8/124	38.7	9.1	4.6–20.9	< 0.01	4.4	2.0–11.1	< 0.01
Nampula/Nacala	116/300	6.5	REF					
**Age group (years)**								
18–24	2/80	2.5	1					
≥25	122/344	35.5	21.4	6.5–131.6	< 0.01	22.8	4.6–414.6	< 0.01
**Gender**								
Male	119/408	29.2	0.9	0.3–2.9	0.86	–	–	–
Female	5/16	31.2	1					
**Marital status**								
Single/never married	73/249	29.3	0.6	0.3–1.1	0.12	0.72	0.4–1.4	0.38
Cohabitating/married	18/89	20.2	0.4	0.2–0.8	< 0.01	1.3	0.7–2.6	0.32
Widowed/divorced/separated	33/86	38.4	1					
**Education level**								
No formal education/Primary school	57/177	32.2	1.2	0.8–1.9	0.27	–	–	–
Secondary/Higher	67/246	27.2	1					
**Age of first drug use**								
< 18	12/49	24.5	1					
18–24	57/202	28.2	1.2	0.6–2.5	0.60	–	–	–
≥25	51/161	31.7	1.4	0.7–3.0	0.34	–	–	–
**No of sexual partners, last 12 months**								
0	27/85	31.8	1					
1	23/83	27.7	0.8	0.4–1.5	0.56	–	–	–
≥2	68/231	29.4	0.9	0.5–1.5	0.68	–	–	–
**Gave money, good or services in exchange for sex in the, last 12 months**^**a**^								
Yes	44/176	25.0	0.6	0.4–1.0	0.07	–	–	–
No	74/223	33.2	1					
**Received money, goods or services in exchange for sex, last 12 months**								
Yes	15/70	21.4	0.6	0.3–1.1	0.10	–	–	–
No	103/329	31.3	REF					
**Ever received drugs in exchange for sex, lifetime**								
Yes	16/57	28.1	0.9	0.4–1.7	0.82	–	–	–
No	108/366	29.5	1					
**Had unprotected sexual intercourse at last sex**								
Yes	38/191	19.9	0.55	0.3–1.0	< 0.05	–	–	–
No, used a condom	86/233	36.9	1.6	0.9–2.7	1.12	–	–	–
Not sexually active			REF					
**Frequency of drug use, last 12 months**								
Did not use	1/22	43.2	1					
Daily	102/236	12.7	15.9	3.3–288.9	< 0.01	–	–	–
Few times per year/month week	21/145	14.5	3.0	0.5–55.9	0.29	–	–	–
**Ever shared needles or syringes**								
Yes	96/236	40.7	3.9	2.5–6.4	< 0.01	4.0	2.1–7.7	< 0.01
No	28/188	14.9	1					
**Shared syringes in the past month**								
Yes	55/133	41.4	2.2	1.4–3.5	< 0.01	0.53	0.4–1.1	0.08
No	69/291	23.7	1					
**Ever injected with a previous used needle/syringe**								
Yes	119/342	34.7	7.9	3.4–23.1	< 0.01	12.1	4.1–46.3	< 0.01
No	5/80	6.2	1					
**Ever shared any other injection equipment**^**b**^								
Yes	45/141	3.19	1.3	0.8–2.1	0.26	–	–	–
No	63/238	26.5	1					
**Had access to brand new syringes in the past 12 months**								
Yes	106/365	29.0	1.1	0.5–1.9	0.75	–	–	–
No	18/58	31.0	1					
**Received condom in the past 12 months**								
Yes	51/165	30.9	1					
No	73/257	28.4	0.8	0.5–1.3	0.58	–	–	–
**Ever incarcerated**								
Yes	98/282	34.8	2.3	1.4–3.9	< 0.01	1.3	0.7–2.4	0.40
No	26/142	18.3	1					

^a^ Excluded female participants

^b^ Other includes spoon, cotton or cleaning agent

## Discussion

Our data indicates that approximately one-third of PWID participants have serological evidence of HIV (44.9%), HBV (32.8%) or HCV (38.3%), which is substantially higher than prevalence rates estimated for PWID reported from sub-Saharan African region for the three infections at 18.3, 3.7, 21.8%, respectively [1].

Our prevalence of HIV/HBV co-infection (13.1%) fall within the range observed in a previous study in the sub-Saharan region (0–28.4%) [27]. The HIV/HBV co-infection estimate is higher than that among other population groups in the Mozambique where there was an estimated 4.9% of youths HIV infected in Maputo [13] and 9.1% of untreated HIV-infected adults [14]. The hepatitis B vaccine was only introduced into the Mozambican national vaccine program for children in 2001 [28]. Although the HBV vaccine is not widely available for the adult population, anti-retroviral therapy (ART) based-on tenofovir (TDF), lamivudine (3TC) and efavirenz (EFV) has dual treatment benefits for both HBV and HIV infections [9, 12, 27].

The prevalence of HIV/HCV co-infection among PWID participants found in this study (29.5%) was substantially higher than the estimated prevalence in the sub-Saharan region (5.7%). Only a few studies have investigated HIV/HCV co-infection among PWID in the sub-Saharan region, and there is no previous empirical estimation HIV/HCV co-infection in Mozambique. Despite the lack information, there is clear evidence that HIV is associated with a higher risk of infection with HCV due to the similar mode of transmission though infected blood [29, 30].

In this study, we found that the prevalence of HIV/HBV and HIV/HCV co-infection among PWID increased with age. Several studies demonstrated that co-infection with HBV or HCV increase with age among HIV-infected individuals [31, 32]. Because HIV and viral hepatitis are chronic and sometimes asymptomatic infections, it is likely that individuals are at an even higher risk of progression to chronicity given the combined effects of age and related immune system dysfunction [33].

Our study also showed that PWID who re-used needle/syringe after someone had injected were almost five and 12 times as likely to be co-infected with HIV/HBV and HIV/HCV, respectively. Other studies have found that HIV and hepatitis transmission are associated with high-risk injection practices such as injection with a syringe previously used by another PWID [2, 9, 10, 30, 34]. Individuals living with HIV/HCV co-infection are less likely to clear acute HCV infection and more likely to transmit the virus which is particularly of concern for PWID who may be sharing used needles [35].

Despite overwhelming evidence of the effectiveness of harm reduction for preventing the spread of HIV and reducing other health issues associated with drug use, global harm reduction service coverage remains insufficient [36]. Mozambique does not have integrated harm reduction programs and policies to reduce the high-risk sexual and drug use behaviors that contribute to the growing HIV epidemic in the country [18]. In 2018, the first drop-in center for people who use drugs was opened as a demonstration site in Maputo to provide comprehensive medical care for people who use drugs before linkage to more comprehensive healthcare services. Clients receive point-of-care screening for HIV, hepatitis, syphilis, tuberculosis and referrals for clinical care as needed [37]. However, so far, the services are restricted to one urban location in the country.

A comprehensive harm reduction package needs to be adopted and should include access to new needle and syringe; opioid substitution therapy (OST); HIV services (including HIV prevention counseling, testing an ART); prevention and treatment of STIs; prevention, diagnosis and treatment of TB and viral hepatitis (B and C), risk reduction information and education for PWID and their sex partners, and condom distribution programmes for PWID and their sex partners [38–40]. Some countries have adopted an integrated harm reduction package to prevent both HIV and hepatitis infection among PWID, given their similar mode of transmission via sexual exposure and high-burden of infection in specific populations [9, 36, 41]. Efforts to promote condom use must specifically address PWID in an environment free to stigma and discrimination [42].

Although this represents the first analysis of HIV a viral hepatitis among PWID in Mozambique, some limitations should be considered. Given the low sample size, this secondary analysis pooled results from the two survey cities resulting in the loss of social networks and chains. As such, these results should be interpreted with caution when generalizing to the full PWID population in these cities. Also, there was a change in recruitment criteria during the survey, to include individuals who ever injected drugs without a prescription, which may have resulted in including participants with lower recent risk of HIV and hepatitis infection. However, since most participants reported having injected during the last month prior to the survey, the change in inclusion criteria may not have had a significant influence. As is the case with all cross-sectional surveys, self-reported data could be subject to recall bias and/or social desirability bias and it is also difficult to assess causality. Finally, many participants refused to have the HCV test (9.4% of the sample), perhaps due an absence of point-of-care testing or treatment options within the national health care system at the time of survey implementation, which may underestimate the true prevalence of HCV in the population.

## Conclusions

This was the first analysis to evaluate the epidemiological profile and risk factors for HIV/HBV and HIV/HBC co-infections among participants in the PWID BBS survey in Mozambique. The high burden of disease among this population requires a enhance public health interventions since HIV and viral hepatitis co-infection increases morbidity and mortality, as well changes the natural history of all infections. An integrated harm reduction strategy is necessary to address all infections simultaneously. These services must be enhanced at health facilities but implementation must also be decentralized through community-based outreach, drop-in centres and special environments, such as prison settings. Further investigation should assess the feasibility of introducing HBV vaccination in this high-risk population within the Mozambican context, as an important strategy prevent associated co-morbidities and improve their overall health status.

## Data Availability

The dataset analyzed for the current study are fully available at the Data Management Unit of the Mozambique National Institute of Health (INS) data repository for researchers who meet the criteria for access to confidential data following concept note submission. For more information can be found in: www.ins.gov.mz or through: secretaria@ins.gov.mz.

## Abbreviations

ART: Antiretroviral therapy
Anti-HCV: Hepatitis C virus antibody
BBS: Biological and behavioral survey
HBsAg: Hepatitis B surface antigen
HBV: Hepatitis B virus
HCV: Hepatitis C virus
HIV: Human immunodeficiency virus
PWID: People who inject drugs
RDS: Respondent-driven sampling
STI: Sexual Transmitted Infections
